# Salmagundi

**Published:** 1886-09

**Authors:** 


### SALMAGUNDI.

EDITED BY E. G. BETTY, D.D.S.

GOOD ADVICE.—Dr. A. H. Thompson, Topeka, Kansas, is always ready with a paper, and he keeps a stock by having a note book at hand in which thoughts that may strike him are noted down and written up at leisure; go thou and do likewise, then you will always have a paper, when called to furnish one.—*Archives of Dentistry.*

DR. W. H. SILLITO, of Xenia, O., called upon us one day to report a walking expedition he had just made in company with a couple of cronies of like ilk. They made the *grande tour* through a number of towns in Southern Ohio, in ten days, covering something like two hundred miles. With the bicyclist, the shot-gun-shootist, and the walkist, to what straits have we come?

INJURIOUS TO THE TEETH.—Many drugs and many diseases exert an injurious influence on the teeth; among the former, iron, alum, citric, nitric, and other acids, and acid solutions of quinine; among the latter, measles, scarlet fever, acid dyspepsia, rickets, septicæmia, syphilis, and diseases of the womb. Even pregnancy tends to impair the teeth; this, however, is supposed to be caused by the demand for bony constituents for the growth of the child.—*Medical World.*

